# Improved cognitive performance in trace amine-associated receptor 5 (TAAR5) knock-out mice

**DOI:** 10.1038/s41598-022-18924-z

**Published:** 2022-08-29

**Authors:** Silvia Maggi, Carlotta Bon, Stefano Gustincich, Valter Tucci, Raul R. Gainetdinov, Stefano Espinoza

**Affiliations:** 1 School of Psychology, University of Nottingham, Nottingham, NG7 2RD UK; 2 Central RNA Laboratory, Istituto Italiano di Tecnologia (IIT), Genova, Italy; 3 Laboratory of Genetics and Epigenetics of Behaviour - Istituto Italiano di Tecnologia, via Morego, 30, 16163 Genova, Italy; 4 Institute of Translational Biomedicine, St. Petersburg State University, St. Petersburg, Russia 199034; 5 St. Petersburg University Hospital, St. Petersburg State University, St. Petersburg, Russia 199034; 6 Department of Health Sciences and Research Center On Autoimmune and Allergic Diseases (CAAD), University of Piemonte Orientale (UPO), Novara, Italy

**Keywords:** Neuroscience, Cognitive neuroscience

## Abstract

Trace amine-associated receptors (TAARs) are a family of G protein-coupled receptors present in mammals in the brain and several peripheral organs. Apart from its olfactory role, TAAR5 is expressed in the major limbic brain areas and regulates brain serotonin functions and emotional behaviours. However, most of its functions remain undiscovered. Given the role of serotonin and limbic regions in some aspects of cognition, we used a temporal decision-making task to unveil a possible role of TAAR5 in cognitive processes. We found that TAAR5 knock-out mice showed a generally better performance due to a reduced number of errors and displayed a greater rate of improvement at the task than WT littermates. However, task-related parameters, such as time accuracy and uncertainty have not changed significantly. Overall, we show that TAAR5 modulates specific domains of cognition, highlighting a new role in brain physiology.

## Introduction

Trace amine-associated receptors (TAARs) are a family of G protein-coupled receptors (GPCR) discovered in 2001^1,2^. TAAR1 is the first member of this family and the most studied one^3,4^. It is expressed in distinct brain regions, and several studies demonstrated its ability to modulate the dopaminergic, serotonergic and glutamatergic systems^5–11^. Initial studies described the other TAARs members as exclusively olfactory receptors sensing innate odours^12^, however, recent evidence shows that most of them are also expressed in the central nervous system (CNS) as well as in the periphery^13–15^. TAAR5 was first discovered in human samples and named putative neurotransmitter receptor (PNR)^16^. Northern blot experiments demonstrated its expression in different brain regions, including the amygdala, caudate nucleus, hippocampus, hypothalamus and thalamus^16^. In mice, in situ hybridization studies confirmed the presence of TAAR5 transcript in the amygdala, the arcuate nucleus and the ventromedial hypothalamus^17^. By using a TAAR5 knock-out line (TAAR5-KO) expressing the LacZ reporter instead of the TAAR5 gene, a distinct expression was observed not only in the olfactory bulb but also in limbic regions such as the amygdala, the entorhinal cortex, the hippocampus, the nucleus accumbens and the thalamic and hypothalamic nuclei^15^.

Olfactory TAAR5 functions are related to its ability to sense trimethylamine (TMA)^12^, a chemical present in mouse urine that is attractive in mice and repulsive in rats and humans^18^. TMA is a product of microbial fermentation of choline and is present in human bodily fluids and certain foodstuffs such as spoiled fish. Interestingly, a recent population study found a rare TAAR5 polymorphism that decreases the aversion to fish odour in carriers^19^. In the brain, TAAR5 modulates emotional behaviour and the serotonergic system^15^. Indeed, TAAR5-KO mice display an anxiolytic and antidepressant-like phenotype. 5-HT1A receptors are more sensitive to agonists, and the level of serotonin and its metabolites are altered in the striatum, the hippocampus and the hypothalamus of TAAR5-KO mice^15^. Conversely, the injection of α-NETA, a TAAR5 agonist, causes a sensorimotor gating deficit in rats and increases mismatch negativity-like response in both rats and mice^20,21^, thus indicating cognitive deficits psychosis-related as seen in humans and experimental animals^22^.

Cognition is a high order function that depends on the proper functioning of several brain areas and involves many neurotransmitter systems. Among others, serotonin is an important player in decision-making and behavioural flexibility^23,24^. Several psychiatric disorders display problems in cognitive processes. In depression, cognitive impairments are considered a core point of the symptomatology and are often persistent after the remission of the depressive status^25^. Cognitive domains often affected in depression are executive functions, working memory and processing speed. Anxiety and stress have a strong impact on cognitive functions, affecting the performances (e.g. students facing an exam) and influencing the decision-making, especially when involving emotional information^26^. Both antidepressant and anxiolytics drugs may be beneficial in treating these types of cognitive impairments in psychiatric disorders, although results are not always positive, given the complexity and the heterogeneous clinical manifestations of these conditions^27–29^.

This study aimed to evaluate the cognitive functions in TAAR5-KO mice since the role of this receptor in emotional behaviour and the serotonin system. To this end, we used a timing task, the switch task, to unveil a possible role of TAAR5 in decision-making and behavioural flexibility. We found that TAAR5-KO mice performed better than WT littermates, by making fewer errors in the task, learning earlier and having a greater rate of improvement over days. Furthermore, TAAR5-KO mice showed lower impulsivity than WT littermates, suggesting that TAAR5 KO mice are more engaged in the task and adapt more flexibly to changes in the environment.

## Results

### The switch task: a temporal decision-making task in the home cage

To investigate the impact of TAAR5 on high cognitive functions, we tested mice on a temporal discrimination task, called the switch task^30^ (Stupplementary Fig. S1a). This task requires a fine judgment of two different temporal signals that last in the range of seconds^31^. The training lasted two weeks after an initial week of pre-training in which the animals familiarised themselves with the operant wall in the home cage. The operant wall consisted of three holes/hoppers (central, left and right) equipped with an infrared beam to detect the nose-poking (NP) activity. Each hole was additionally supplied with a light bulb on the top to signal the start, the duration and the end of every trial^32^. During the first week of training, the mice had to learn to discriminate between two intervals: one of 3 s (short signal) and the other of 9 s (long signal). Each time interval was associated with one of the two lateral hoppers, and its duration was signalled by the light above the hopper. Short and long signals were randomly intermixed. During the second week, probe trials were introduced with a probability of 20% on each side. Probe trials consisted of a light signal of the same duration, but no reward was delivered in case of a correct response. Probe trials were randomly intermixed with regular trials aiming to assess the accuracy in time perception and the perseverance in nose-poking activity around the learned target time.

**Figure 1:**
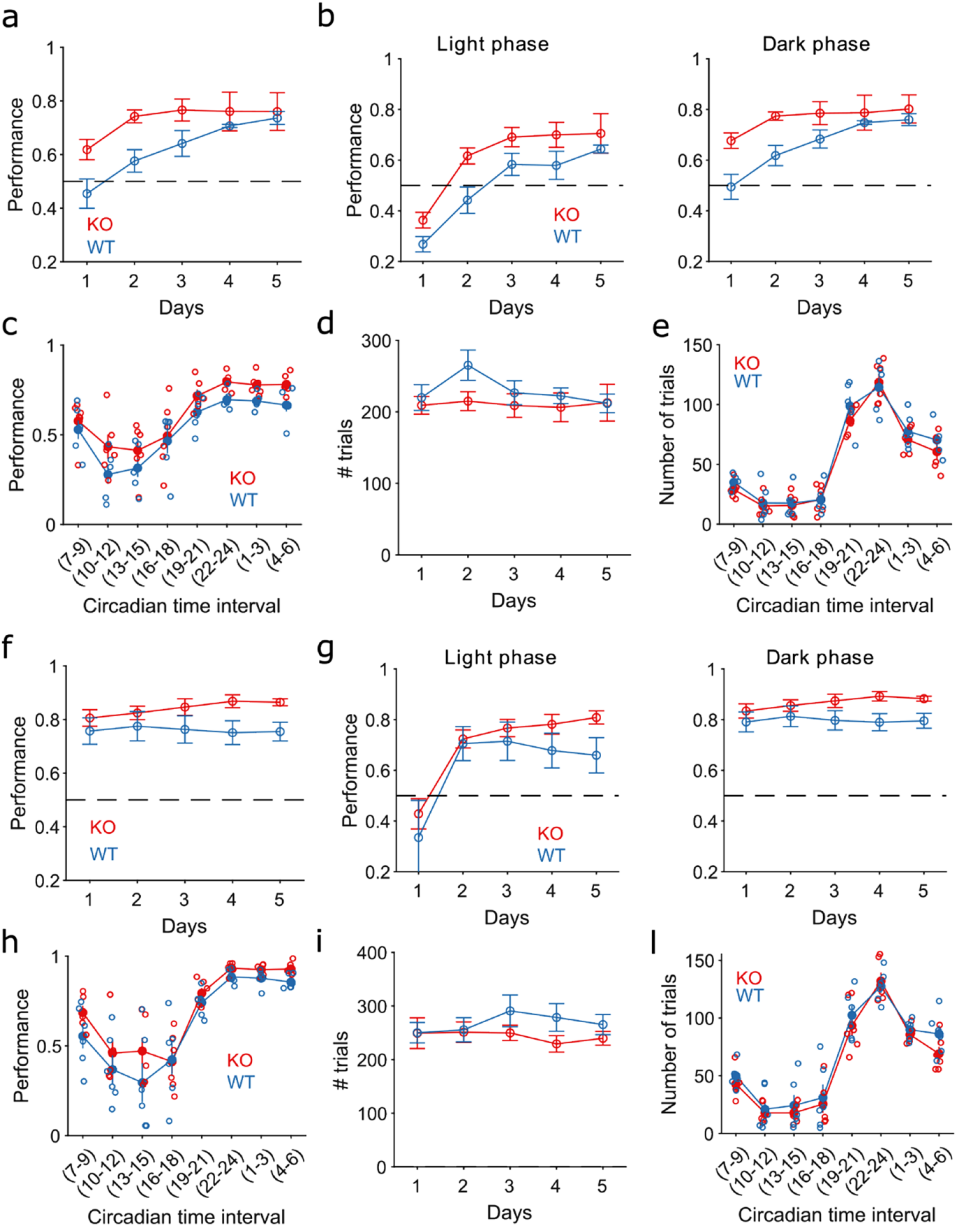
TAAR5 KO mice performance is higher than WT. (**a-e**). week 1. f-l. week 2. (**a**). Performance over days for KO mice (red) and WT (blue). Performance is the ratio of correct trials over the total number of trials per day. Correct trials include rewarded and probe trials. (2-way Anova, effect of time *p* < 0.005, F = 6.78, effect of group p < 0.005, F = 13.23). Every panel shows mean ± SEM b. Performance per day during the light (left panel) and dark (right panel) phases. Curves show mean ± SEM (2-way Anova, light phase: effect of time *p* < 0.005, F = 20.71, effect of group *p* < 0.005, F = 15.08; dark phase: effect of time *p* < 0.005, F7.22, effect of group *p* < 0.005, F = 16.01). (**c**). Performance along the circadian cycle grouped by intervals of 3 h. Each empty circle is a subject (2-way Anova, effect of time *p* < 0.005, F = 23.87, effect of group *p* < 0.005, F = 14.54) (**d**). Number of trials over days of training (2-way Anova, non significant effect of time *p* = 0.4, F = 0.88, no effect of group *p* = 0.09, F = 2.94). (**e**). Total number of trials per hour along the circadian cycle grouped by intervals of 3 h. (2-way Anova, effect of time *p* < 0.005, F = 103.26, no effect of group *p* = 0.1, F = 2.69). (**f**). Similar to panel a for week 2. (2-way Anova, no effect of time *p* = 0.9, F = 0.19, effect of group *p* < 0.005, F = 11.54). (**g**). Similar to panel b for week 2. (2-way Anova, light phase: effect of time *p* < 0.005, F = 9.83, no effect of group *p* = 0.06, F = 3.5; dark phase no effect of time *p* = 0.8, F = 0.29, effect of group *p* < 0.005, F = 13.7). (**h**). Similar to panel c for week 2. (2-way Anova, effect of time *p* < 0.005, F = 34.42, effect of group *p* = 0.008, F = 7.32). (**i)**. Similar to panel d for week 2. (2-way Anova, no effect of time *p* = 0.8, F = 0.29, no effect of group *p* = 0.07, F = 3.22). (**l**). Similar to panel e for week 2. (2-way Anova, effect of time *p* < 0.005, F = 71.4, no effect of group *p* = 0.08, F = 3.1).

The main advantage of this task is that it allows monitoring the animals’ behaviour in their home cage continuously for several days (24 h a day, for several weeks), reducing animal stress and highlighting subtle differences even between mouse substrains^32^. This advantage allows to obtain statistically powerful results with a reduced number of animals. We first checked whether the TAAR5-KO mice showed alteration or deviation of their circadian parameters compared to WT mice. We couldn’t observe any differences between the groups in terms of the circadian period (Supplemental Fig. S1b-e) or activity distribution along the 24 h cycle (Supplemental Fig. S1d-g) in both weeks of training. A significant effect of time was present in the first week of training (Supplemental Fig. S1c), likely due to a learning dynamic that required higher activity at the beginning of the training to obtain enough food. This activity decreased over the training with a performance improvement, and it increased again in the second week, where it remained constant (Supplemental Fig. S1f). The increase during the second week was due to the addition of probe unrewarded trials, which required higher engagement in the task to obtain the same amount of food. The continuous monitoring of mice performance allowed to track the evolution over time and the learning of fine cognitive functions. With this task, we expected to identify when and how a change in the cognitive performance of TAAR5-KO mice emerged during training.

### TAAR5-KO mice show better performance over training

In a previous study, it was found that TAAR5-KO mice showed less anxiety and antidepressant-like phenotype^15^. Here, we asked whether this alteration in emotional behaviour affected fine cognitive functions and potentially contributed to a better performance in temporal decision-making tasks. Indeed, we observed that KO mice showed on average to perform better for the duration of the entire training (Fig. 1). This improved performance was evident since the first day of training, reaching a steady performance by day 2 for KO mice (Fig. 1a). In contrast, WT mice showed a significantly lower performance (Fig. 1a–f). We further explored whether this effect was specific to the light or the dark phase. We found that in both phases and for the entire duration of the training, KO mice showed a better performance compared to WT (Fig. 1b,g). A more temporally refined analysis revealed that this difference was maintained hourly over the circadian cycle (Fig. 1c,h). We already excluded that the better performance was related to altered activity patterns (hyper- or hypo-activity), as shown in Supplementary Fig. S1. Furthermore, this difference could not be related to different food intake and feeding behaviour as both groups received a comparable number of pellets and performed a comparable number of trials on a daily and hourly basis (Fig. 1d, e, i, l; Supplementary Fig. S2).

The better performance of TAAR5-KO mice and the comparable number of trials between the two groups suggests that the difference should be in the number of rewarded trials (performance includes probe trials too. Probe trials are correct trials but not rewarded). However, the absolute number of rewarded trials was not different along the circadian cycle between the two groups (Supplementary Fig. S2a–d), suggesting that both groups received the same amount of food. Instead, the difference resided in the reward rate over days (Fig. 1a, Supplementary Fig. S2b–e) and was partially observable along the circadian cycle too (Fig. 1c, Supplementary Fig. S2c–f), suggesting that TAAR5-KO mice were proportionally more efficient at the task despite the number of overall trials and reward received was similar between the two groups. Furthermore, we found proportionally more probe trials for TAAR5-KO mice compared to their littermate control (Supplementary Fig. S2g,h,i), confirming that the better performance of TAAR5-KO mice was due to a combination of successfully rewarded trials and accurately performed trials. The efficiency of TAAR5-KO mice could depend on two factors. One is that they make fewer mistakes and potentially learn faster, suggesting that they are more efficient and engaged in the task. The other factor is task-related and assumes that TAAR5-KO mice are more accurate in temporal decision-making tasks. We explored here both possibilities.

### TAAR5-KO mice make fewer mistakes and have a higher rate of improvement

We hypothesised that TAAR5-KO mice were performing better due to a general non-task-related cognitive ability to learn better and remain engaged in the task for a longer time. Therefore, we first evaluated the error trials to see where these mice were performing better. Then, we examined the learning phase and rate of improvement to investigate whether they were learning earlier or were consistently improving more than WT.

**Figure 2:**
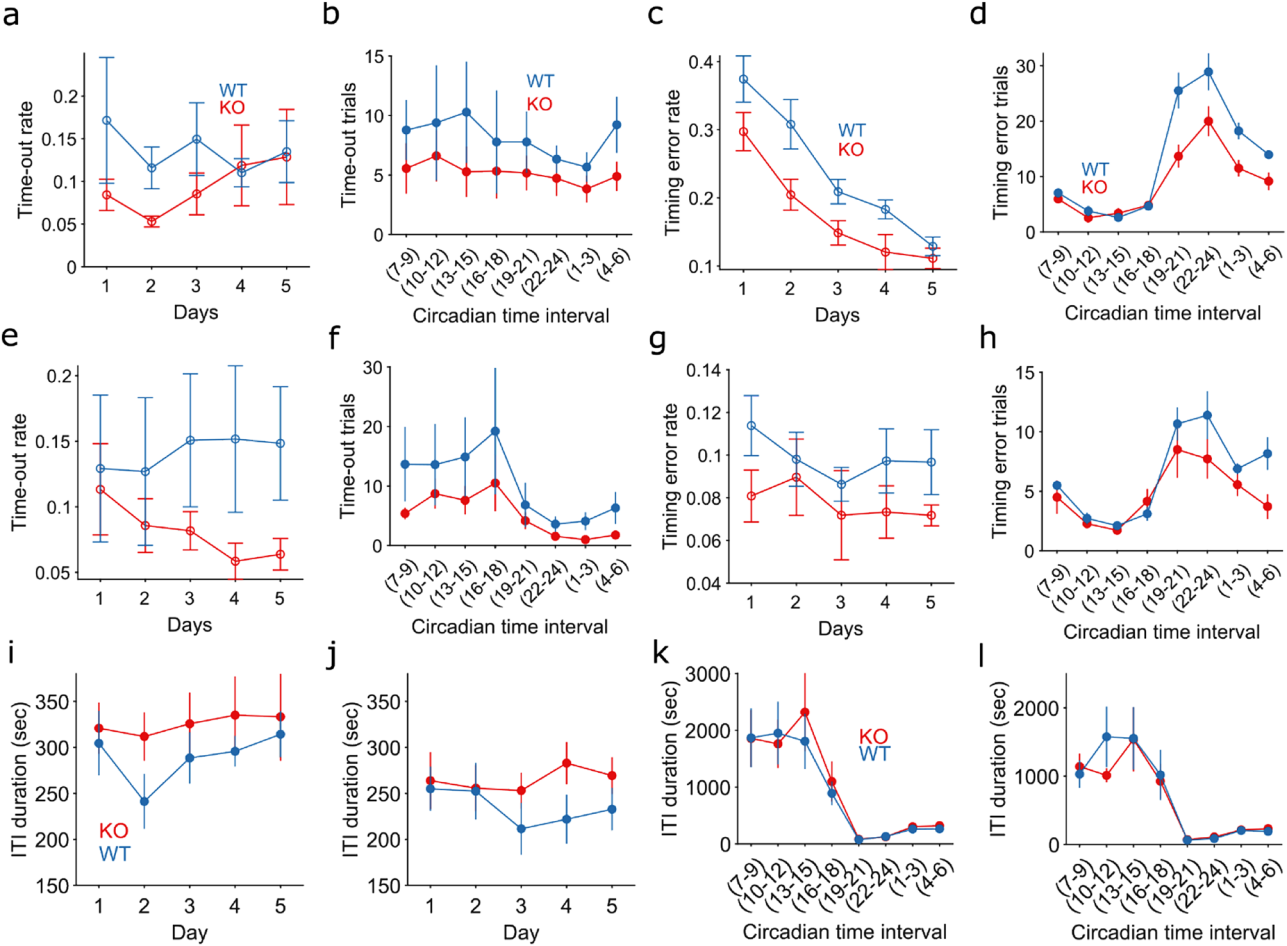
TAAR5 mice make fewer errors. (**a-d**). These panels refer to the first week of training. (**e–h**). Refer to the second week of training. Every panel shows mean ± SEM. Blue symbols are for wild-type mice, red symbols for KO mice. 2-way ANOVA has been used for every panel. (**a**). Time-out trial rate over the first week of training (no effect of time *p* = 0.77, F = 0.44 or group *p* = 0.09, F = 2.85). (**b**). Absolute number of time-out trials over the circadian cycle for three hours interval (no effect of time *p* = 0.92, F = 0.36, effect of group *p* = 0.02, F = 5.27). (**c**). Timing error rate over days of training (significant effect of time *p* < 0.005, F = 26.86 and effect of group *p* < 0.005, F = 18.23). (**d**). Absolute number of timing error trials over the circadian cycle (significant effect of time *p* < 0.005, F = 45.16, group *p* < 0.005, F = 24.56 and interaction *p* < 0.005, F = 3.71). (**e**). Similar to panel a. for the second week of training (no effect of time *p* = 0.99, F = 0.07, significant effect of group *p* = 0.02, F = 5.75). (**f**). Similar to panel b for second week of training (significant effect of time *p* = 0.03, F = 2.23, significant effect of group *p* = 0.02, F = 5.63). (**g**). Similar to panel c. (no effect of time *p* = 0.68, F = 0.57, significant effect of group *p* = 0.02, F = 5.61) (**h**). Similar to panel d (2-way ANOVA: significant effect of time *p* < 0.005, F = 12.01 and effect of group *p* = 0.01, F = 6.79). (**i**). Average ITI duration for each day during the first week of training (no effect of time *p* = 0.8, F = 0.38, or group *p* = 0.06, F = 3.53). (**j**). Similar to panel i for second week of training (no effect of time *p* = 0.6, F = 0.62, or group *p* = 0.08, F = 3.17). (**k**). ITI duration along the circadian cycle for intervals of three hours. (Significant effect of time *p* < 0.005, F = 12.56, no effect of group *p* = 0.6, F = 0.25.) (**l**). Similar to panel k for second week of training. (Significant effect of time *p* < 0.005, F = 12.56, no effect of group *p* = 0.6, F = 1.2).

Indeed, TAAR5-KO mice made fewer errors over both training days and the circadian cycle (Fig. 2, Supplementary Fig. S3 a, b, e, f). To further explore the type of errors most frequently made by WT mice, we separated the analysis into time-out and timing error trials. Time-out trials ended without any response, as the maximum time window allowed to nose-poke in one of the locations to make a choice elapsed without response. Timing error trials are instead those in which the animal made the incorrect choice. We found fewer time-out trials over the circadian cycle (Fig. 2 b, f) and over the two weeks of training (Fig. 2a, e) for TAAR5-KO mice, suggesting that these mice are overall more engaged in the task. Indeed, KO mice complete their trials by making choices much more often than WT, and this is also true during the light phase when animals are sleepier. However, the reduced number of time-out trials might also suggest impulsivity in KO mice. To exclude this hypothesis, we analysed the intertrial interval (ITI). If TAAR5-KO mice were more impulsive, we should expect shorter ITI. Instead, we observed no difference between WT and KO mice in the average ITI duration on a daily (Fig. 2 i,j) and hourly (Fig. 2 k,l) basis.

The timing error trials were significantly different between groups over days of training and the circadian cycle (Fig. 2c, d, g, h, Supplementary Fig. S3d, h). In particular, TAAR5-KO mice were more accurate (less timing error trials) than WT, especially during the dark phase, when animals are more active (Fig. 2d, h).

**Figure 3:**
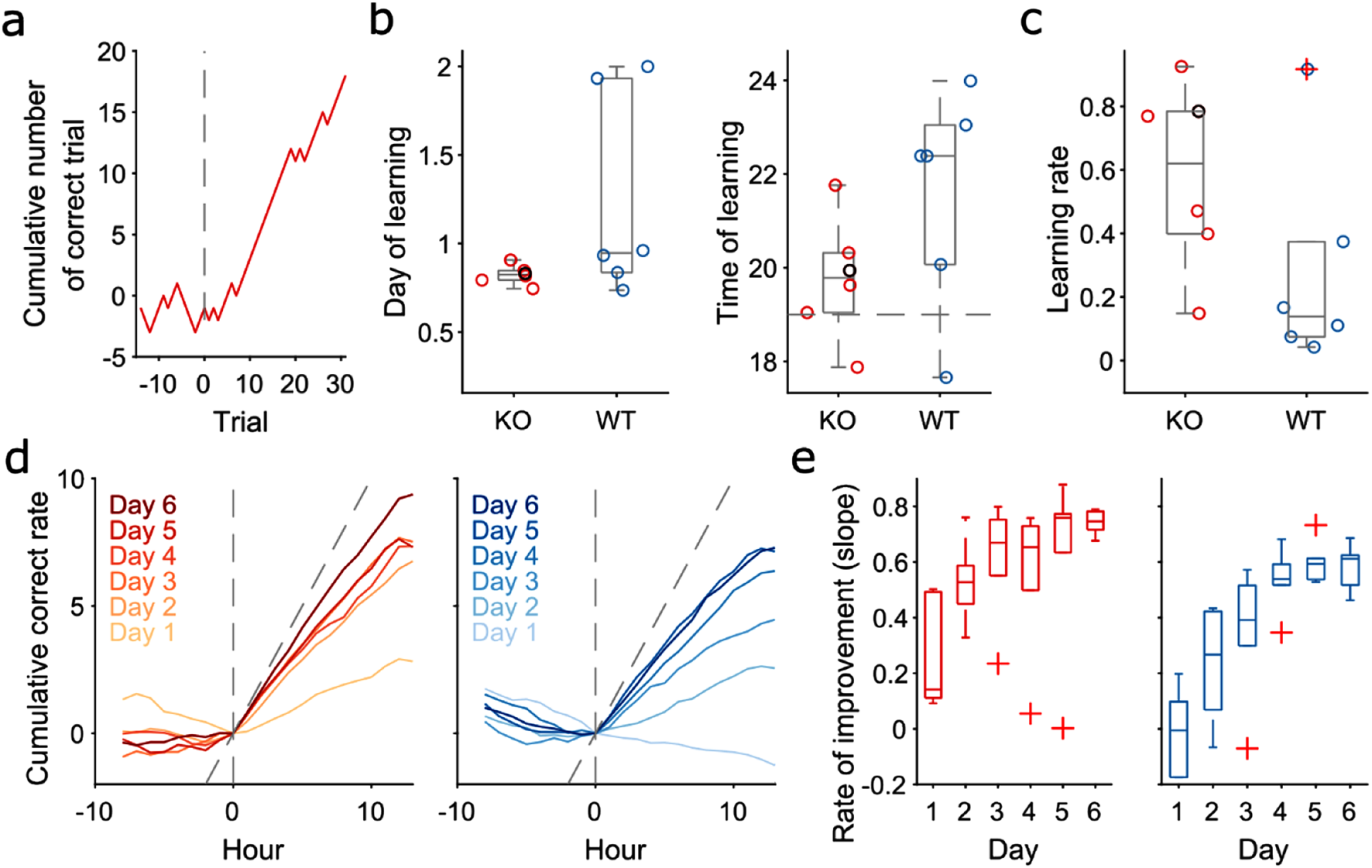
TAAR5 mice improve at a higher speed. (**a**). Cumulative number of correct trials for an example subject around the learning trial (trial 0). This curve increases (+ 1) when correct trial and decreases (1) when error. For each subject, we identified the learning trial (trial 0) as the trial with maximum inflexion between the curve before and after the trial. Vertical dashed line identifies the learning trial for this example subject. (**b**). The day (left panel) and time (right panel) of the learning trial for each subject. Boxplot distribution shows median, interquartile and extreme value of the distribution. Each circle is a subject, and the black circle is the example subject in panel a. Horizontal dashed line in the right panel identifies the start of the dark phase. (**c**). Comparison of the learning rate between KO and WT mice. Each circle is a subject. The learning rate was measured as the difference between the slopes of the regression curves before and after the leaning trial (panel a). (**d**). Cumulative correct rate over days for each hour around the light off (hour zero). Vertical dashed line identifies the time of light off; diagonal dashed line identifies the optimal correct rate (where all the trials are correct). Red curves are KO mice, blue curves are WT mice. The color shade reflects the days of training (light color = first day, darker color = last day). (**e**). The slope of the regression curves after the time of light off in panel d identifies the rate of improvement over days for each group. (2-way ANOVA: significant effect of time *p* < 0.005, F = 13.58 and group *p* < 0.005, F = 15.45).

To further investigate when and how the improvement in the performance emerged in TAAR5-KO mice, we analyzed the learning during the first week of training to identify its occurrence. Multiple factors could determine the higher performance over time: either an earlier and better learning or a better rate of improvement over time or a combination of both. We defined learning as the time point (or trial) at which the cumulative number of correct trials (Supplementary Fig. S3i) showed the maximum inflexion (Fig. 3a, see Methods). We found that both groups learned early in training; KO mice within the first day and all WT within the second day (Fig. 3b), with no significant difference between groups. They also learned at the beginning of the dark phase, when mice become more active (Fig. 3b, right panel). The learning trial and time of learning were not significantly different between groups. Neither was the learning rate, defined as the change in the slopes of the regressions between before and after the learning point (Fig. 3c, see Methods), despite a marginal trend suggesting better improvement by TAAR5-KO mice (Kolmogorov–Smirnov test, *p* = 0.07).

The above definition of learning is somewhat arbitrary. Therefore, we tested our hypothesis with an alternative definition of learning as the trial after which the animal performed at or above 80% correct for at least 20 consecutive trials. We found similar results in terms of day of learning (Supplementary Fig. S3k) and a significant difference in terms of time of learning (Supplementray Fig. S3l), with KO-mice learning at the beginning of the dark phase compared to WT.

Since TAAR5-KO mice learn as well as WT and at the same time, then we hypothesised that TAAR5-KO mice might show a higher rate of improvement than WT over time. Therefore, we computed the correct rate (see Methods) per hour over the training (Supplementary Figure S3j). We observed a clear circadian effect resulting in a steeper correct rate during the dark phases compared to the light phases. We compared the average correct rates around the time the light is turned off (hour 0 in Fig. 3d) over days, and we observed an increasingly steep correct rate for both groups over days. However, TAAR5 KO mice showed to get close to the optimal correct rate (the diagonal dashed line, Fig. 3d) faster and earlier. To further compare the distributions of correct rates across subjects and over time, we quantified the slope of the cumulative correct rate curve after the light switch for each subject in each day of training. We found a significant effect of group and time, suggesting that TAAR5 mice reached a better performance sooner and their rate of improvement was also higher. These results support the hypothesis that the performance improvement is not task-related but instead is due to a higher engagement (lower time-out trials) and better cognitive flexibility (higher rate of improvement). However, the significantly lower timing error rate compared to the time-out rate (Supplementary Fig. S3c,d,g,h) did not exclude that TAAR5-KO mice might show significant alteration in timing parameters. We explored this option below.

### Interval-timing is preserved in TAAR5-KO mice

**Figure 4:**
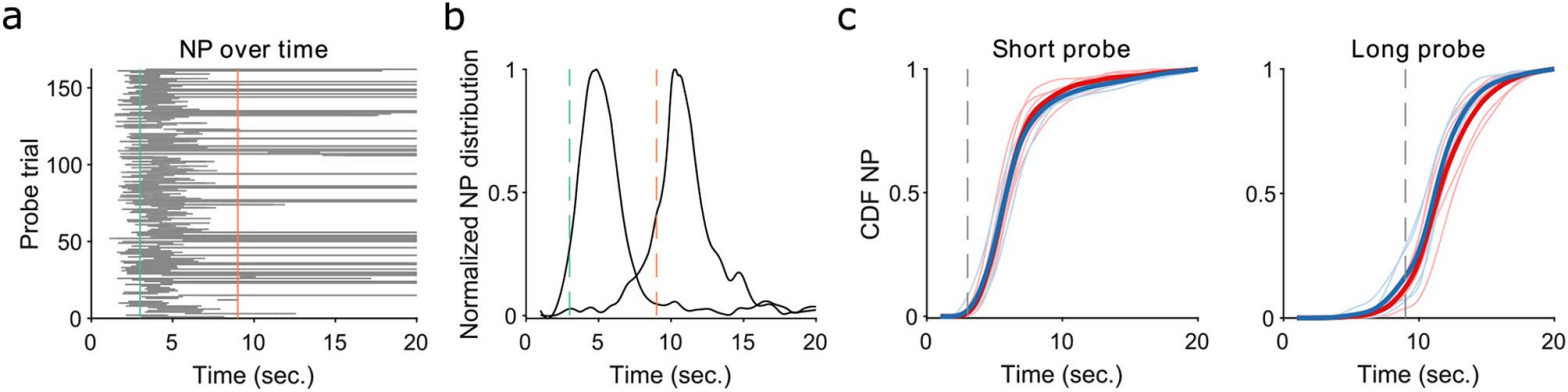
Preserved Interval timing in TAAR5-KO mice. (**a**). Example distribution of NP during short probe trials. Each row is a probe, the solid grey line identifies the interval in which the animal was poking into the short hopper location. Green and orange vertical lines identify the short and long time, respectively. (**b**). Normalised NP distribution for short and long probe trials for the example subject in a. Green and orange vertical dashed lines identify the short and long time, respectively. (**c**). Cumulative distribution of NP activity for each subject and each group. Solid lines identify group average. Each single lighter line is a subject. Blue curves are for WT mice, while red curves for TAAR5-KO, as before.

To fully explore the efficiency of KO mice in this behavioural paradigm, we checked the accuracy in task-related parameters. In particular, probe trials were introduced during the second week of training to investigate the persistence in nose-poke activity around the target time when the reward was not delivered. Two possibilities of poking behaviour are feasible. Prolonged poking might suggest perseveration in reward-seeking, whereas reduced poking activity suggests inactivity, which we excluded (Supplementary Fig. S1) or inattention. To explore the former, we evaluated the distribution of nose-pokes during probe trials for short (Fig. 4a,b) and long duration (Fig. 4b) trials. No difference in the distributions of nose-poke activity between TAAR5 KO and WT mice (Fig. 4c) for either short or long probes has been found, suggesting that TAAR5 KO mice have intact interval-timing estimation.

### TAAR5-KO mice have optimal temporal accuracy

**Figure 5:**
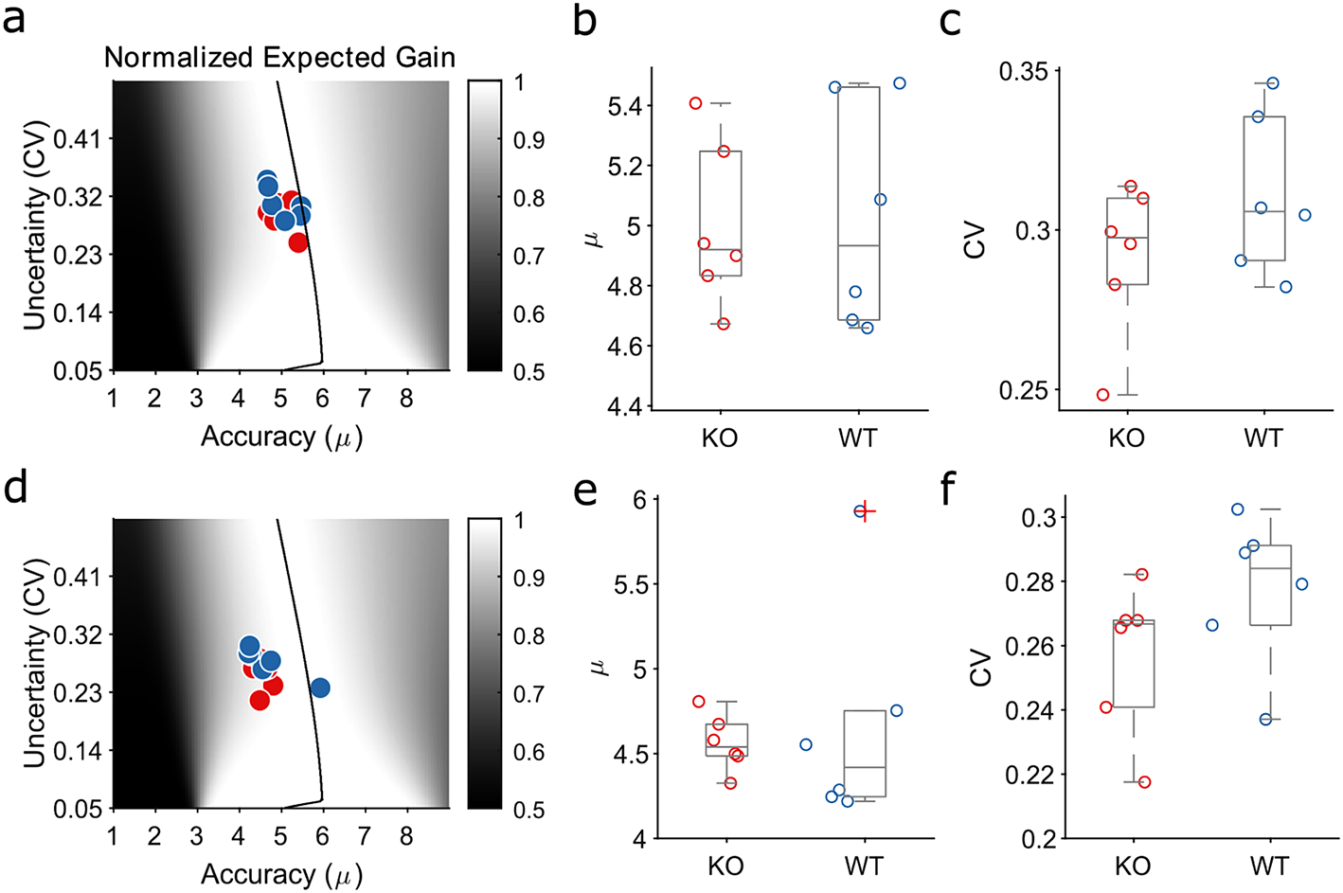
Preserved temporal decision-making in TAAR5 mice. (**a**). Normalized expected gain surface. Shades of grey identify the expected gain given every combination of accuracy (μ) and uncertainty (CV). The solid black line identifies the maximum expected gain for each combination of accuracy and uncertainty. Blue (WT) and red (KO) dots show the distribution of expected gain for each subject during the first week of training. (**b**). Boxplot distribution of accuracy (μ) during the first week of training. Each circle is a subject. Kolmogorov–Smirnov test *p* = 0.8 (**c**). Boxplot distribution of uncertainty (CV) during the first week of training. Each circle is a subject. Kolmogorov–Smirnov test *p* = 0.8 (**d**). Similar to panel a for the second week of training. (**e–f**). Similar to panels b-c, respectively, for the second week of training. Kolmogorov–Smirnov test, *p* = 0.3 and *p* = 0.3, respectively for panel (**e**) and (**f**).

Relevant task-related parameters of the switch task include estimating temporal accuracy and uncertainty (see Methods). Typically, control animals develop an optimal strategy to solve this task moving to the short location soon after self-initiating the trial and waiting until the short time elapses. If the light signal is short, the animal pokes in the short location; otherwise, it switches to the long location, waiting for the long duration to elapse. The time of the switch from short to long location is called ‘switch latency’. By knowing the distribution of the switch latencies, we can estimate how close the behaviour is to an optimal one^33^ (see Methods). For each subject, we assessed the switch latency distribution parameters from the fitted normal distribution^41^. The average switch latency reflects the subject’s target switch latencies, also called timing accuracy (μ), while the dispersion around the mean (the coefficient of variation, CV = σ/μ) reflects the endogenous timing uncertainty. We estimated the accuracy and uncertainty of every subject during the first (Fig. 5 top panels) and second (Fig. 5 bottom panels) week of training. Both groups were performing nearly perfect (Fig. 5a, d) since no difference in the distributions of the parameters between TAAR5-KO and WT mice (Fig. 5b, c, e, f) has been found. These results suggest that TAAR5-KO mice performance is not related to the specific task demands but instead is a general feature of this mouse line, likely to show consistent performance improvement across a wide range of behavioural and cognitive tasks.

## Discussion

TAAR5 expression in the CNS was demonstrated in recent studies, and some of its putative functions in brain physiology were characterised^13–15,34^. TAAR5 is involved in regulating emotional behaviour, and TAAR5-KO mice show an anxiolytic and anti-depressant-like phenotype^15^. In this study, we evaluated the role of TAAR5 on cognitive processes and we showed that TAAR5-KO mice were able to perform better by making fewer errors and displaying a higher rate of improvement in the performance. In particular, we found that TAAR5-KO mice were more engaged (lower time-out trials) in the task, especially during the light phase, where mice are typically sleepier, and were more accurate (lower timing error) especially during the dark phase. This improvement in the performance was not due to earlier learning, but instead, we found a constant higher rate of improvement throughout the training.

Apart from its role in olfaction, the comprehension of TAAR5 functions in brain physiology is still in its infancy. Since its low expression, initial reports did not find TAAR5 outside the olfactory epithelium^12^. However, independent reports show a discrete TAAR5 expression in several brain areas using different techniques, demonstrating its presence in limbic regions such as the amygdala, entorhinal cortex, nucleus accumbens, thalamic and hypothalamic nuclei^13–15,17^. Recently, by analysing transcriptomic datasets derived from human samples, it was found a low but ubiquitous expression of TAAR5 in limbic and cortical areas^13,14^. A similar situation was initially in TAAR1 studies since it was found at low levels in discrete brain regions^3,35^. However, TAAR1-KO mice display a clear phenotype and many reports demonstrated its relevant role in dopamine, serotonin and glutamate homeostasis^8,9^. TAAR1 selective agonists are now in late-stage clinical trials with the indication of potential antipsychotic agents^36^.

TAAR5-KO mice did not show gross abnormalities nor overt neurological phenotype^15^. However, a series of behavioural tests assessing emotional behaviour highlighted that TAAR5-KO mice are less anxious and with an antidepressant-like phenotype compared to WT littermates^15^. Serotonin and its metabolites levels are also altered in this mouse line and the hypothermic effect of the 5-HT1A agonist 8-OH-DPAT is increased. Another report shows that striatal dopamine levels and the number of dopamine neurons in the substantia nigra is increased and, interestingly, the neurogenesis in the subventricular and subgranular zones is increased in mutants^37^. Recently, an altered sensorimotor function in TAAR5-KO mice was also demonstrated^31^. Some of these previously observed alterations might suggest that changes in performance could occur due to an increase eager to reward rather than enhanced cognitive flexibility. However, we showed that WT and KO mice performed a comparable number of trials (Fig. 1d,e,i,l), number of rewarded trials (Supplementary Fig. S2a,d) and had a similar distribution of reaction times (Supplementary Fig. S3m, Methods). These results exclude a motivational state to reward as a potential contributor to the changes observed.

To unveil the possible roles of TAAR5 in cognition, we used a home cage behavioural paradigm that tested temporal decision-making in mice. Home cage behavioural test allows to collect large amount of data and to reveal subtle differences between substrain of animals^32^. In this study, this paradigm highlighted several interesting aspects in the analysis of cumulative correct rate (Fig. 3d) over the days of training at the time the light switched off. First, both genotypes show a clear step-change in the average hourly activity between the light and dark phases. Second, before the light switch off (before 0 in Fig. 3d) KO and WT mice have a 50% or lower probability of success, respectively, over the days of training, with no effect of time. This phenomenon suggests a sleepiness effect unrelated to the level of training. Third, as soon as the light switch off (after trial 0 in Fig. 3d), we can observe a clear improvement in the performance, which increases over the training days. Finally, we showed that TAAR5-KO mice had, on average, a better rate of improvement across all training days (Fig. 3e).

In this test, animals have to learn the task to obtain a food pellet, in particular, to discriminate between two-time intervals. Both WT and TAAR5-KO learned quite fast the test (Fig. 3). The speed in learning is due to the continuous exposure to the task (24/7), forcing the animal to work to obtain food in their home cage. This removes the stress caused by moving the animal from one cage to another and allows mice to engage in the task at their own rhythm, which typically follows a circadian oscillation through the 24 h.

Interestingly, KO mice performed better in the test, indicating an increased accuracy in the decision-making process visible from the lower timing error trials (Fig. 2). The better performance was also evident in the light phase, a period of the day where animals are usually sleepier and make more errors. Indeed, WT mice showed higher time-out trials. These trials occur when the animal self-initiates the trial but is not keen to complete it. The elevated number of time-out trials during the light phase suggests that these trials do not happen due to a momentary inattention to the task but are more likely due to disengagement and sleepiness.

Another interesting distinction between time-out trials and timing error trials is their temporal evolution. In particular, the latter reflects the learning dynamic; the former is constant over the entire training. Figure 2c shows the decrease in timing error trials over the first week of training, which is then maintained constant throughout the second week (Fig. 2g). This dynamic nicely resembles the improvement and maintenance of the performance seen in Fig. 1a, f. On the contrary, time-out trials remain constant over the entire training, supporting our previous claim that these trials are a reflection of sleepiness.

If we look at task-related parameters, both groups of mice displayed a correct interval-timing estimation and an optimal combination of temporal accuracy and uncertainty. Overall, our results suggest that TAAR5-KO mice are better learners, more engaged in the task, and adapt more flexibly to change in the environment. This overall better performance is not related to the specific task demand but it may be a general feature of these mice.

To confirm these data, more behavioural tests specific to each cognitive domain are needed to understand the precise role of TAAR5 in cognition. It should be noted that these subtle differences may be difficult to unveil using standard tests done during a few hours in the daylight phase. Cognitive assays usually reveal more easily deficits rather than a pro-cognitive effect in WT animals. Another option would be to inject a TAAR5 antagonist to mimic these actions in vivo, similarly to what has been done for TAAR1 studies. Although the first TAAR5 antagonists were described some years ago^38^, no other selective and potent compounds have been reported so far. In principle, a selective TAAR5 antagonist may be a new potential drug with several therapeutic indications with a new mechanism of action. Apart from the endogenous agonist TMA that has a clear role in olfaction, only another putative agonist has been found and tested in animal models, namely α-NETA. Interestingly, in mice and rats, this compound was able to induce psychotic-like behavioural abnormalities, including features related to cognitive deficits present in psychotic patients^20,21^.

How TAAR5 influences cognitive domains is still under investigation. A recent report show that TAAR5-KO mice have an increased number of dopaminergic neurons in SNpc and an increased levels of dopamine in the striatum^37^. The correct levels of dopamine and dopamine signaling is fundamental for correct cognitive functions. Altered levels of dopamine in pathological conditions, such as in Parkinson’s Disease and Schizophrenia lead to cognitive deficits^39^. Thus, further studies are needed to understand the importance of dopamine alterations in TAAR5-KO mice in the cognitive alterations seen in our behavioral paradigm. Serotonin, a neurotransmitter whose levels are altered in TAAR5-KO mice, plays an interesting role in cognition^23,24^. Although the serotonergic system is very complex and serotonergic receptors are a big family of GPCRs with a myriad of functions, there is a general consensus that serotonin is important in decision making. Moreover, compounds that increase serotonergic transmission, such as antidepressants, are used in neuropsychiatric diseases where cognitive impairments are present and in particular impairment in decision making^23^. 5-HT1A agonists, especially ones acting mostly on post-synaptic receptors, increase behavioural flexibility and facilitate performances^40^. Similarly, anxiolytics may be beneficial in decision making, particularly when the decision is influenced by an emotional component^26^. An anxiolytic effect may also facilitate a flexible choice behaviour, increasing the speed of finding the optimal strategy. TAAR5-KO mice display a decrease total content of serotonin in the striatum and the hippocampus^15^. However, there are no data on the extracellular serotonin levels or the elecrophysiological properties of the serotonergic neurons in these mice. A recent study showed that TAAR5-KO mice had increased adult neurogenesis in both the subventricular zone and the subgranular zone^37^. Adult neurogenesis is linked to many aspects of brain physiology, including cognition and the behavioural effect of stress and antidepressant^41^. In particular, several pieces of evidence suggest a role of adult neurogenesis in cognitive flexibility and that this action may reduce anxiety and depressive-like behaviour^41^.

In conclusion, we showed that TAAR5 might be considered a new player in cognitive processes and a potential new drug target for various neuropsychiatric disorders involving deficiencies in emotional states and cognition.

## Materials and methods

### Mice and husbandry

Groups of 8–12 weeks old male mice were studied (TAAR5-KO and their WT littermates). Each group included 6 mice and were generated as described previously^15^. All mice were group-housed two weeks before the experiment with food and water ad libitum under a 12:12 light–dark cycle (lights on from 7:00 to 19:00). The week before the experiment, 20 mg food pellets were gradually mixed into regular food for habituation. Then mice were singly housed in type III TSE PhenoMaster cages (TSE Systems Bad Homburg, Germany) and subjected to the experimental phases. The animal study was reviewed and approved by all procedures involving animals. Their care was carried out in accordance with the guidelines established by the European Community Council (Directive 2010/63/EU of September 22, 2010) and was approved by the Italian Ministry of Health. During the experimental phases, animal wellbeing was monitored daily. If the weight loss was between 10 and 20% (referring to the free-feeding weight taken on day one), one or two additional standard food pellets (approx. 1.3 g) were given, respectively. If weight loss exceeded 20% of the free-feeding weight, animals had to be culled. All the animals in this study completed the experiment.

### Apparatus and procedure

In this study, we used an automated operant wall (Cognition and Welfare, COWE), developed by TSE Systems (Germany) based on its PhenoMaster System. The device consists of three holes/hoppers over a metal wall inserted in type III cages. Each hole is equipped with infrared beams that detect the nose poking. A LED with 4 mcd (millicandela) of luminous intensity is mounted in each hopper to serve as a stimulus. The two lateral hoppers are attached to independent hidden feeders that dispense 20 mg dustless precision pellets (BioServ, USA). The sensors (LED and infrared beams) and the actuator (feeder) were remotely controlled via computer to design trial by-trial protocols for individual and/or group cages. Each COWE cage (n = 12) was maintained in individual ventilated and sound-proof light-controlled cubicles. The house light (approximately 100–110 lx) was timed on a 12:12 light–dark schedule as described above.

### Experimental design

The whole experiment consisted of two experimental phases following a pre-training phase. The experiment included 12 animals. During the pre-training phase, all mice familiarised themselves with the COWE cage to obtain food pellets from both lateral hoppers. This pre-training phase consisted of self-initiating trials by nose-poking in the central hopper triggering the switch-on of the lights in the three hoppers. Nose-poking in the lateral hoppers gave access to food rewards. No temporal limitations were imposed during this phase, and the goal of the pre-training phase was to develop the association between hopper location and food pellet. The trial ended when the animal received a pellet from each side and concomitantly lights switched off. Each trial was followed by an intertrial interval (ITI). The ITI was set as a 30 s fixed delay plus a random interval drawn from a geometric distribution with a mean of 60 s. The mice could not initiate a new trial during the ITI.

After four days of pre-training, all mice were introduced to the two consecutive experimental phases, each lasting about a week. During the first week, mice were trained in the switch task^32^. In this task, animals had to discriminate the duration of two light signals (i.e., short- vs. long-latency signals, called here short and long trials) to obtain a food pellet in a trial. The duration of the light signal determined the location of the pellet availability. Short (T_S_) and long (T_L_) trials were randomly intermixed with the same probability (P(T_S_) = P(T_L_) = 0.5).

The left hopper was associated with the short trials, whereas the right hopper was associated with the long trials. The first nose poke after the short or long duration at the corresponding lateral hopper was reinforced with a food pellet, the trial was declared finished and the ITI started. Any nose-poke at the long location after a short signal or vice-versa was not reinforced, triggering the start of the ITI. These trials were classified as timing error trials. If the animal self-initiated the trial but did not engage in the task by not poking in the hoppers, the trial ended after 30 s with no reward. These trials were classified as time-out trials. Short-latency signals lasted 3 s and long-latency signals lasted 9 s.

In the second week of training, we introduced 20% probe trials for both short-latency and long-latency trials. This means that short probes (Sp) and long probes (Lp) were introduced with the same conditional probabilities P(Sp|T_S_) = P(Lp|T_L_) = 0.2. During probe trials, the signal was presented as for regular trials but the correct responses of the animals were not reinforced. Probe trials lasted 30 s each, followed by an ITI as described earlier in this section. This manipulation allowed us to further characterise mouse timed behaviour in its full complexity.

### Data analysis

We recorded all events in the COWE cages with a millisecond resolution and these events were timestamped. Each timestamp was paired with an event code that identifies a specific type of event (i.e., light on/off, nose in/out, etc.). This strategy allowed us to standardise specific codes for data analysis across laboratories^32^. Every analysis was performed using MATLAB (www.mathworks.it) software.

All the analyses were performed on the first and second weeks of training separately to highlight the impact of probe trials. The analyses along the circadian cycles were computed for each subject by quantifying the parameter (e.g. performance, time-out trials, etc.) for each hour and then averaged across three-hour intervals. From the resulting dataset, we computed a group average. The performance for each mouse was computed as the count of correct trials over the total number of trials performed. Correct trials included all rewarded trials during the first week of training; however, during the second week of training, correct trials included also probe trials.

We computed the cumulative number of correct trials to identify the learning point for each subject. This curve is the cumulative sum of correct (+ 1) and error (-1) trials over time. To identify the learning trial, we fitted a piecewise linear regression model to each trial allowing a minimum of five trials from the beginning of training. The model was applied to a moving window of length twenty trials, moving every five trials. The model comprised a robust regression line fitted to the cumulative curve before each trial and another line fitted to the cumulative curve after the trial. The learning trial was then identified as the first trial having the maximum increase in slope between the fitted regression before and after the trial. The day of learning and time of learning was reconstructed from the timestamps of the identified learning trial. The learning rate is the difference between the slope of the regression line before and after the learning trial.

To quantify the rate of improvement across days during the transition between light and dark phase, we quantified the cumulative correct rate curve for each subject. This curve looked at an interval of 22 h around the time of the switch-off of lights (10 h before and 12 h after the switch-off of the house light). For each hour, each subject and each day, we computed the rate of correct trials.

To assess the circadian rhythmicity, we quantified the circadian period with a non-linear curve-fitting to the number of nose-pokes over the recording hour. The periodic function that was fit to these data is defined by Eq. (1).

1 $$F\left( {T,K} \right) = A \sin \left( {\frac{2\pi }{P}T + \Phi } \right) + A$$

where T = (t1, . . . , tn) are the time points (15 min) during the recording and K = (A, P, Φ) are, respectively the amplitude, period, and phase of the sinusoidal function. Best-fit coefficients (A, P, Φ) were determined by minimising the mean-square difference between F and the data. The fit was repeated for multiple values of the parameter P (from 21 to 27 h every 0.5 h). The goodness of the fit was quantified by the Pearson Correlation Coefficient (CC) between the data of each subject and the corresponding fit function F. The best fit for P converged to the Subjective Period (Supplementary Fig. 1b) for every initialisation of the parameter P between 22 and 26 h for every subject.

Probe trials were introduced to assess how timing behaviour changed when correct responses were reinforced probabilistically (note that these probabilities were equal between the two trial types). The raster plot of NP activity (Fig. 4a) for each subject and each trial type was analysed. We showed the empirical normalised NP distribution for an example subject (Fig. 4b) and compared the cumulative distribution of NP between groups (Fig. 4c).

We assessed the temporal decision-making performance by analysing the switch latency or accuracy (Fig. 5). The switch-latency is defined only for long trials and is the trial time at which the mouse leaves the short-latency location for the long-latency location^42^. For each subject, the distribution of the switch latencies was fit with a Gaussian function. From each Gaussian fit, we estimated the mean (μ) and variance (σ). We considered the μ as the accuracy in timing estimation and the coefficient of variation (CV = μ/ σ), which is the dispersion of switch latencies around the mean, as the timing uncertainty^43^. The dependence of optimal target-switch latency on the level of timing uncertainty was formulated in^42^ and then expanded in^32^. This formulation, called the Normalized Expected Gain function (Eq. 2), allows the evaluation of timed behaviours within the framework of optimality based on experienced probabilistic reinforcement, endogenous timing uncertainty, and the payoff matrix.

2 $${\varvec{EG}}\left( {\hat{\user2{t}}} \right) \propto 1 - {{\varvec{\Phi}}}\left( {{\varvec{T}}_{{\varvec{S}}} ,\hat{\user2{\mu }},\hat{\user2{\sigma }}} \right) + {{\varvec{\Phi}}}\left( {{\varvec{T}}_{{\varvec{L}}} ,\hat{\user2{\mu }},\hat{\user2{\sigma }}} \right)$$

The optimal target switch latency for a given mouse was defined as the $\widehat{{\varvec{\mu}}}$ that maximises the output of Eq. 2, where Φ is the normal cumulative distribution function. We computed the reaction time for each trial as the time elapsed between the end of the short or long signal and the first nose-poke to the left or right hopper, respectively. For each subject, we estimated the empirical probability distribution function (PDF in Supplementary Fig. 3 m) and compared the mean ± SEM between the two groups.

### Statistical analysis

Data were analysed with a one-way or repeated measure ANOVA test using the Matlab package. The significant difference and F-statistic, the ratio of the mean squares, are reported for every test in the Figure legends.

### Ethical approval

The animal study was reviewed and approved by all procedures involving animals and their care were carried out in accordance with the guidelines established by the European Community Council (Directive 2010/63/EU of September 22, 2010) and were approved by the Italian Ministry of Health. The study is reported in accordance with ARRIVE guidelines.

## Supplementary Information

Supplementary Information.

## Data Availability

A GitHub repository containing the data and the Matlab codes to reproduce the main findings of this paper is available at https://github.com/sibangi/TAAR5_repo.

## References

[1] Bunzow JR, Sonders MS, Arttamangkul S, Harrison LM, Zhang G, Quigley DI, Darland T, Suchland KL, Pasumamula S, Kennedy JL, Olson SB, Magenis RE, Amara SG, Grandy DK (2001). Amphetamine, 3,4-methylenedioxymethamphetamine, lysergic acid diethylamide, and metabolites of the catecholamine neurotransmitters are agonists of a rat trace amine receptor. Mol. Pharmacol..

[2] Borowsky B, Adham N, Jones KA, Raddatz R, Artymyshyn R, Ogozalek KL, Durkin MM, Lakhlani PP, Bonini JA, Pathirana S, Boyle N, Pu X, Kouranova E, Lichtblau H, Ochoa FY, Branchek TA, Gerald C (2001). Trace amines: Identification of a family of mammalian G protein-coupled receptors. Proc. Natl. Acad. Sci. USA.

[3] Gainetdinov RR, Hoener MC, Berry MD (2018). Trace amines and their receptors. Pharmacol. Rev..

[4] Schwartz MD, Canales JJ, Zucchi R, Espinoza S, Sukhanov I, Gainetdinov RR (2018). Trace amine-associated receptor 1: A multimodal therapeutic target for neuropsychiatric diseases. Expert Opin. Ther. Targets.

[5] Sukhanov I, Caffino L, Efimova EV, Espinoza S, Sotnikova TD, Cervo L, Fumagalli F, Gainetdinov RR (2016). Increased context-dependent conditioning to amphetamine in mice lacking TAAR1. Pharmacol Res..

[6] Pei, Y., Lee, J. A., Leo, D., Gainetdinov, R. R., Hoener, M. C., Canales, J. J. Activation of the Trace Amine-Associated Receptor 1 Prevents Relapse to Cocaine Seeking. *Neuropsychopharmacology* (2014). 10.1038/npp.2014.88 PMC4138750 24722355

[7] Espinoza S, Salahpour A, Masri B, Sotnikova TD, Messa M, Barak LS, Caron MG, Gainetdinov RR (2011). Functional interaction between trace amine-associated receptor 1 and dopamine D2 receptor. Mol. Pharmacol..

[8] Lindemann L, Meyer CA, Jeanneau K, Bradaia A, Ozmen L, Bluethmann H, Bettler B, Wettstein JG, Borroni E, Moreau JL, Hoener MC (2008). Trace amine-associated receptor 1 modulates dopaminergic activity. J. Pharmacol. Exp. Ther..

[9] Wolinsky TD, Swanson CJ, Smith KE, Zhong H, Borowsky B, Seeman P, Branchek T, Gerald CP (2007). The Trace Amine 1 receptor knockout mouse: An animal model with relevance to schizophrenia. Genes Brain Behav..

[10] Revel FG, Moreau JL, Gainetdinov RR, Bradaia A, Sotnikova TD, Mory R, Durkin S, Zbinden KG, Norcross R, Meyer CA, Metzler V, Chaboz S, Ozmen L, Trube G, Pouzet B, Bettler B, Caron MG, Wettstein JG, Hoener MC (2011). TAAR1 activation modulates monoaminergic neurotransmission, preventing hyperdopaminergic and hypoglutamatergic activity. Proc. Natl. Acad. Sci.USA.

[11] Revel FG, Moreau JL, Pouzet B, Mory R, Bradaia A, Buchy D, Metzler V, Chaboz S, Groebke Zbinden K, Galley G, Norcross RD, Tuerck D, Bruns A, Morairty SR, Kilduff TS, Wallace TL, Risterucci C, Wettstein JG, Hoener MC (2013). A new perspective for schizophrenia: TAAR1 agonists reveal antipsychotic- and antidepressant-like activity, improve cognition and control body weight. Mol. Psychiatry.

[12] Liberles SD, Buck LB (2006). A second class of chemosensory receptors in the olfactory epithelium. Nature.

[13] Vaganova, A. N., Murtazina, R. Z., Shemyakova, T. S., Prjibelski, A. D., Katolikova, N. V., Gainetdinov, R. R. Pattern of TAAR5 expression in the human brain based on transcriptome datasets analysis. *Int J Mol Sci* **22**(16).(2021) 10.3390/ijms22168802 PMC8395715 34445502

[14] Gaudel, F., Guiraudie-Capraz, G., Feron, F. Limbic expression of mRNA coding for chemoreceptors in human brain-lessons from Brain Atlases. *Int J Mol Sci* **22**(13), (2021) 10.3390/ijms22136858 PMC8267617 34202385

[15] Espinoza S, Sukhanov I, Efimova EV, Kozlova A, Antonova KA, Illiano P, Leo D, Merkulyeva N, Kalinina D, Musienko P, Rocchi A, Mus L, Sotnikova TD, Gainetdinov RR (2020). Trace amine-associated receptor 5 provides olfactory input into limbic brain areas and modulates emotional behaviors and serotonin transmission. Front. Mol. Neurosci..

[16] Zeng Z, Fan P, Rand E, Kyaw H, Su K, Madike V, Carter KC, Li Y (1998). Cloning of a putative human neurotransmitter receptor expressed in skeletal muscle and brain. Biochem. Biophys. Res. Commun..

[17] Dinter J, Muhlhaus J, Wienchol CL, Yi CX, Nurnberg D, Morin S, Gruters A, Kohrle J, Schoneberg T, Tschop M, Krude H, Kleinau G, Biebermann H (2015). Inverse agonistic action of 3-iodothyronamine at the human trace amine-associated receptor. PLoS One.

[18] Li Q, Korzan WJ, Ferrero DM, Chang RB, Roy DS, Buchi M, Lemon JK, Kaur AW, Stowers L, Fendt M, Liberles SD (2013). Synchronous evolution of an odor biosynthesis pathway and behavioral response. Curr Biol.

[19] Gisladottir RS, Ivarsdottir EV, Helgason A, Jonsson L, Hannesdottir NK, Rutsdottir G, Arnadottir GA, Skuladottir A, Jonsson BA, Norddahl GL, Ulfarsson MO, Helgason H, Halldorsson BV, Nawaz MS, Tragante V, Sveinbjornsson G, Thorgeirsson T, Oddsson A, Kristjansson RP, Bjornsdottir G, Thorgeirsson G, Jonsdottir I, Holm H, Gudbjartsson DF, Thorsteinsdottir U, Stefansson H, Sulem P, Stefansson K (2020). Sequence variants in TAAR5 and other loci affect human odor perception and naming. Curr. Biol..

[20] Aleksandrov AA, Dmitrieva ES, Volnova AB, Knyazeva VM, Polyakova NV, Ptukha MA, Gainetdinov RR (2019). Effect of alpha-NETA on auditory event related potentials in sensory gating study paradigm in mice. Neurosci. Lett..

[21] Aleksandrov AA, Knyazeva VM, Volnova AB, Dmitrieva ES, Korenkova O, Espinoza S, Gerasimov A, Gainetdinov RR (2018). Identification of TAAR5 agonist activity of alpha-NETA and its effect on mismatch negativity amplitude in awake rats. Neurotox. Res..

[22] Light GA, Swerdlow NR (2015). Future clinical uses of neurophysiological biomarkers to predict and monitor treatment response for schizophrenia. Ann. NY Acad. Sci..

[23] Homberg JR (2012). Serotonin and decision making processes. Neurosci. Biobehav. Rev..

[24] Bacque-Cazenave, J., Bharatiya, R., Barriere, G., Delbecque, J. P., Bouguiyoud, N., Di Giovanni, G., Cattaert, D., De Deurwaerdere, P. Serotonin in animal cognition and behavior. *Int J Mol Sci,* **21**(5) (2020) 10.3390/ijms21051649 PMC7084567 32121267

[25] LeMoult J, Gotlib IH (2019). Depression: A cognitive perspective. Clin. Psychol. Rev..

[26] Robinson OJ, Vytal K, Cornwell BR, Grillon C (2013). The impact of anxiety upon cognition: Perspectives from human threat of shock studies. Front. Hum. Neurosci..

[27] Prado CE, Watt S, Crowe SF (2018). A meta-analysis of the effects of antidepressants on cognitive functioning in depressed and non-depressed samples. Neuropsychol. Rev..

[28] Perini G, Cotta Ramusino M, Sinforiani E, Bernini S, Petrachi R, Costa A (2019). Cognitive impairment in depression: Recent advances and novel treatments. Neuropsychiatr. Dis. Treat..

[29] Silver JA, Hughes JD, Bornstein RA, Beversdorf DQ (2004). Effect of anxiolytics on cognitive flexibility in problem solving. Cogn. Behav. Neurol..

[30] Maggi S, Balzani E, Lassi G, Garcia-Garcia C, Plano A, Espinoza S, Mus L, Tinarelli F, Nolan PM, Gainetdinov RR, Balci F, Nieus T, Tucci V (2017). The after-hours circadian mutant has reduced phenotypic plasticity in behaviors at multiple timescales and in sleep homeostasis. Sci. Rep..

[31] Balci F, Papachristos EB, Gallistel CR, Brunner D, Gibson J, Shumyatsky GP (2008). Interval timing in genetically modified mice: A simple paradigm. Genes Brain Behav..

[32] Maggi S, Garbugino L, Heise I, Nieus T, Balcı F, Wells S, Tocchini-Valentini GP, Mandillo S, Nolan PM, Tucci V (2014). A cross-laboratory investigation of timing endophenotypes in mouse behavior. Timing Time Percept..

[33] Balci F, Freestone D, Simen P, Desouza L, Cohen JD, Holmes P (2011). Optimal temporal risk assessment. Front Integr. Neurosci..

[34] Kalinina DS, Ptukha MA, Goriainova AV, Merkulyeva NS, Kozlova AA, Murtazina RZ, Shemiakova TS, Kuvarzin SR, Vaganova AN, Volnova AB, Gainetdinov RR, Musienko PE (2021). Role of the trace amine associated receptor 5 (TAAR5) in the sensorimotor functions. Sci. Rep..

[35] Lindemann L, Hoener MC (2005). A renaissance in trace amines inspired by a novel GPCR family. Trends Pharmacol. Sci..

[36] Koblan KS, Kent J, Hopkins SC, Krystal JH, Cheng H, Goldman R, Loebel A (2020). A Non-D2-receptor-binding drug for the treatment of Schizophrenia. N Engl J Med.

[37] Efimova EV, Kozlova AA, Razenkova V, Katolikova NV, Antonova KA, Sotnikova TD, Merkulyeva NS, Veshchitskii AS, Kalinina DS, Korzhevskii DE, Musienko PE, Kanov EV, Gainetdinov RR (2021). Increased dopamine transmission and adult neurogenesis in trace amine-associated receptor 5 (TAAR5) knockout mice. Neuropharmacology.

[38] Cichero, E., Espinoza, S., Tonelli, M., Franchini, S., Gerasimov, A. S., Sorbi, C., Gainetdinov, R. R., Brasili, L., Fossa, P. A homology modelling-driven study leading to the discovery of the first mouse trace amine-associated receptor 5 (TAAR5) antagonists. *MedChemComm* (**2016**)

[39] Nieoullon A (2002). Dopamine and the regulation of cognition and attention. Prog. Neurobiol..

[40] Alvarez BD, Morales CA, Amodeo DA (2021). Impact of specific serotonin receptor modulation on behavioral flexibility. Pharmacol. Biochem. Behav..

[41] Anacker C, Hen R (2017). Adult hippocampal neurogenesis and cognitive flexibility - linking memory and mood. Nat. Rev. Neurosci..

[42] Balci F, Freestone D, Gallistel CR (2009). Risk assessment in man and mouse. Proc. Natl. Acad. Sci. USA.

[43] Simen P, Balci F, de Souza L, Cohen JD, Holmes P (2011). A model of interval timing by neural integration. J. Neurosci..

