# Supplementary material for: Improved cognitive performance in trace amine-associated receptor 5 (TAAR5) knock-out mice
Source: Sci Rep. 2022 Aug 29;12:14708. doi: 10.1038/s41598-022-18924-z (PMC9424310; doi:10.1038/s41598-022-18924-z)
Supplement: Supplementary file 1 — Supplementary Information. [file 41598_2022_18924_MOESM1_ESM.docx]

**Supplementary Figures**


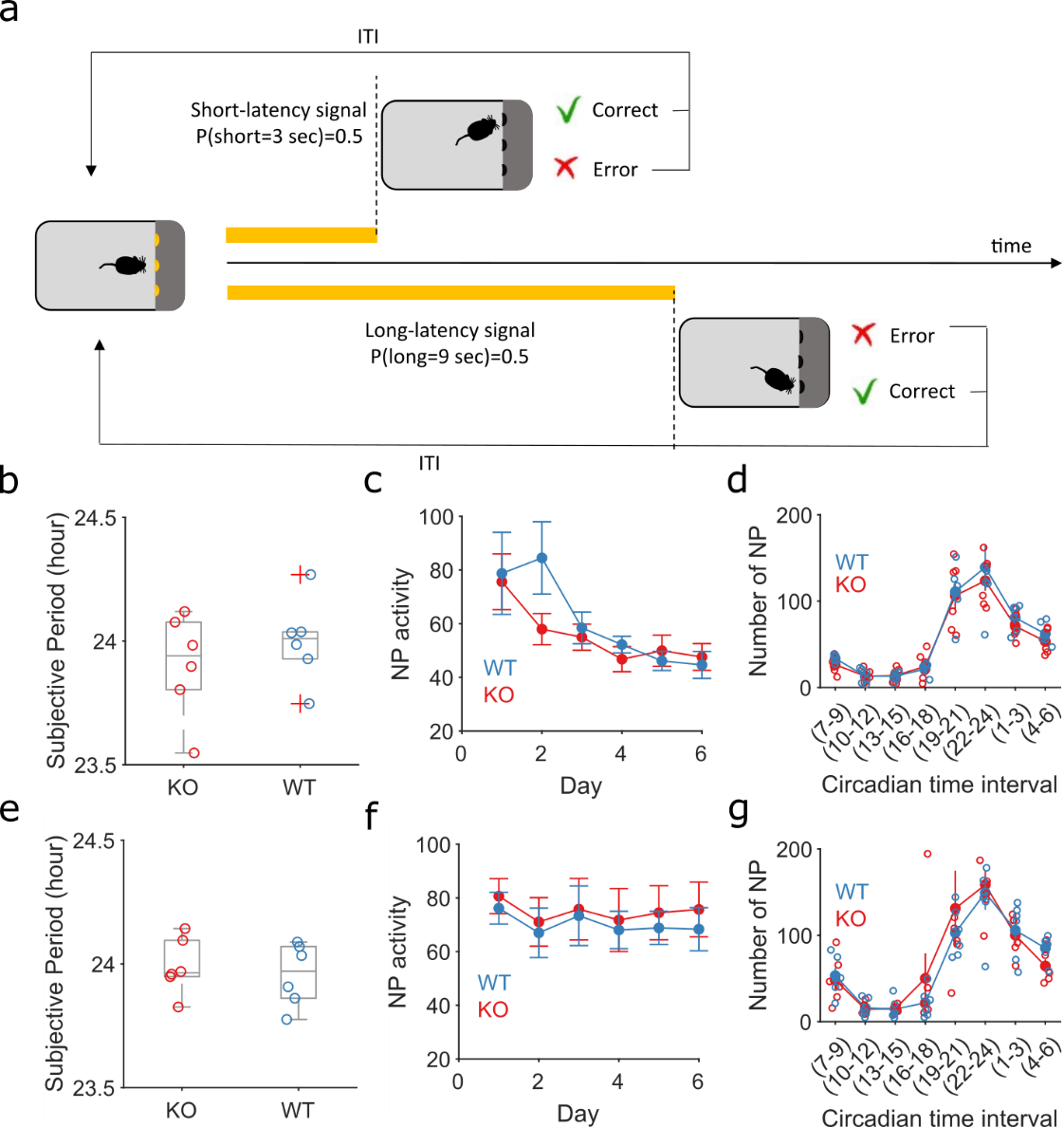


**Supplementary Figure 1: Circadian activity over training. a.** Schematic representation of the task. The probability of short (S) and long (L) signal was 50%. The duration of the short and long signal was 3 and 9 seconds, respectively. **b-d.** Refers to the first week of training. **e-g.** Refers to the second week of training. **b,e.** Boxplot distribution of circadian period for each group. Boxplots shows median (horizontal line within the box), interquartile range (grey box) and outliers (red pluses). Each dot is a subject (n=6 WT and n=6 KO). **c,f.** Average ± SEM number of nose-pokes per hour for each day of training. In panel **c** there is a significant effect of time(2-way ANOVA, p<0.001 F=5.55), but not group (2-way ANOVA, p=0.2 F=1.36). No effects in panel **f** (2-way ANOVA, effect of time p=0.9, F=0.28; effect of group p=0.38, F0.77) . **d-g.** Mean ± SEM nose-poke per hour across the circadian cycle displayed as intervals of three hours. Each empty circle is a subject. Significant effect of time but not group in both weeks (2-way ANOVA. Week 1, effect of time p<0.001, F=43.01; effect of group p=0.2, F=1.17. Week2, effect of time p<0.001, F=19.45; effect of group p=0.6, F=0.27).


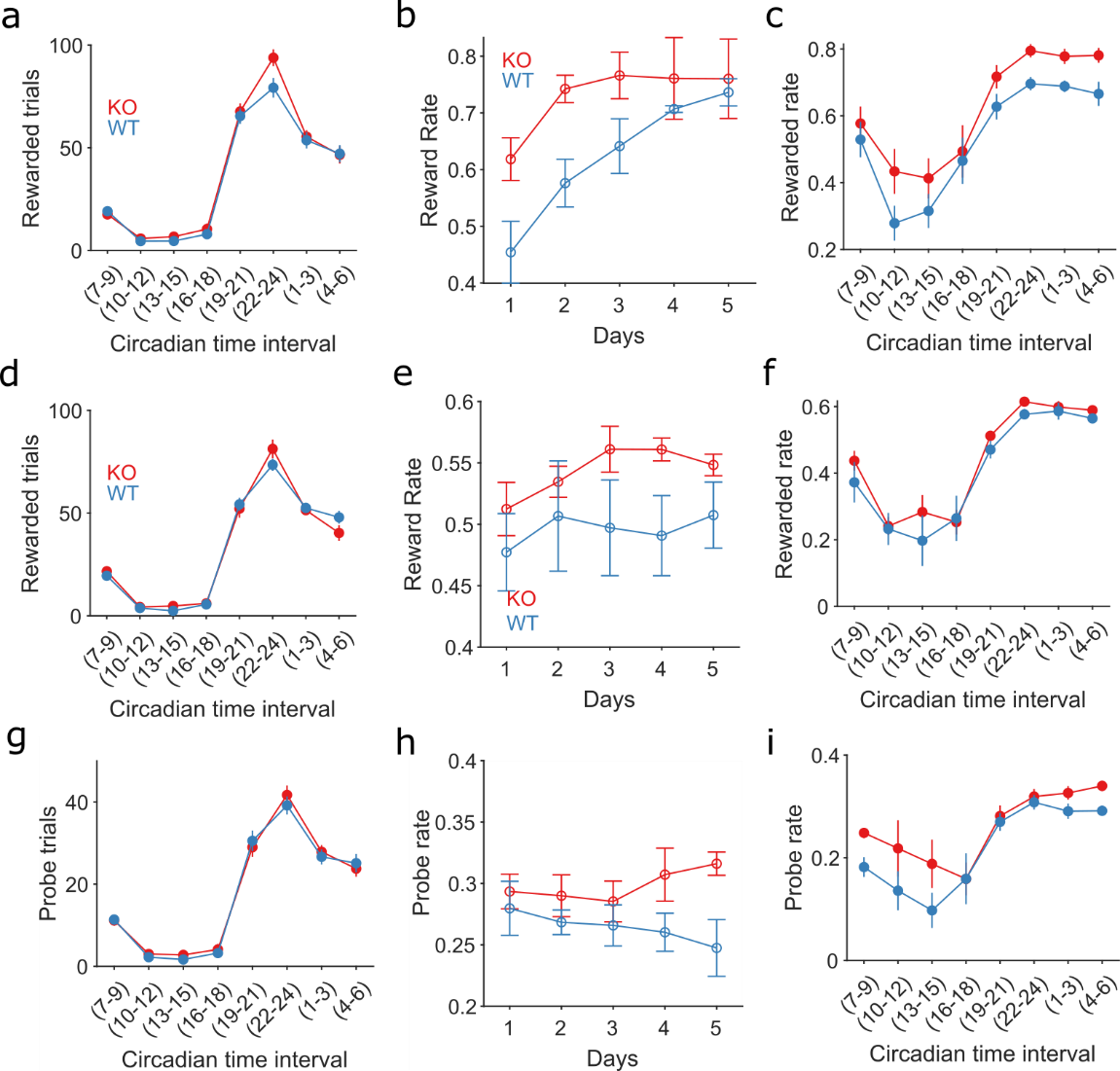


**Supplementary Figure 2: KO mice perform a higher proportion of correct trials. a.** Number of rewarded trials per hour along the circadian cycle grouped by intervals of three hours during week 1. Mean ± SEM for each group. KO mice (red) and WT (blue). Rewarded trials include correct trials without probes. 2-way Anova, effect of time p<0.005, F=206.86, no effect of group p=0.07, F=3.3. **b.** Reward rate per day during the first week of training. 2-way Anova, effect of time p<0.005, F=6.78, effect of group p<0.005, F=13.23. **c.** Reward rate per hour along the circadian cycle grouped by intervals of three hours during week 1. 2-way Anova, effect of time p<0.005, F=23.87, effect of group p<0.005, F=14.54. **d.** Similar to panel a for week 2. 2-way Anova, effect of time p<0.005, F=222.88, no effect of group p=0.8, F=0.06. **e.** Similar to panel b for week 2. 2-way Anova no effect of time,p=0.7, F=0.55, effect of group p=0.008, F=7.6. **f.** Similar to panel c for week 2. 2-way Anova, effect of time p<0.005, F=34.1, no effect of group p=0.09, F=2.86. **g.** Number of probe trials per hour along the circadian cycle during week 2. 2-way Anova, effect of time p<0.005, F=157.33, no effect of group p=0.6, F=0.27. **h.** Probe rate per day during the second week of training. 2-way Anova, no effect of time p=0.9, F=0.12, effect of group p<0.005, F=9.82. **i.** Proportion of probe trial per hour along the circadian cycle grouped by interval of three hours during week 2. 2-way Anova, effect of time p<0.005, F=12.73, effect of group p<0.005, F=8.84.

**
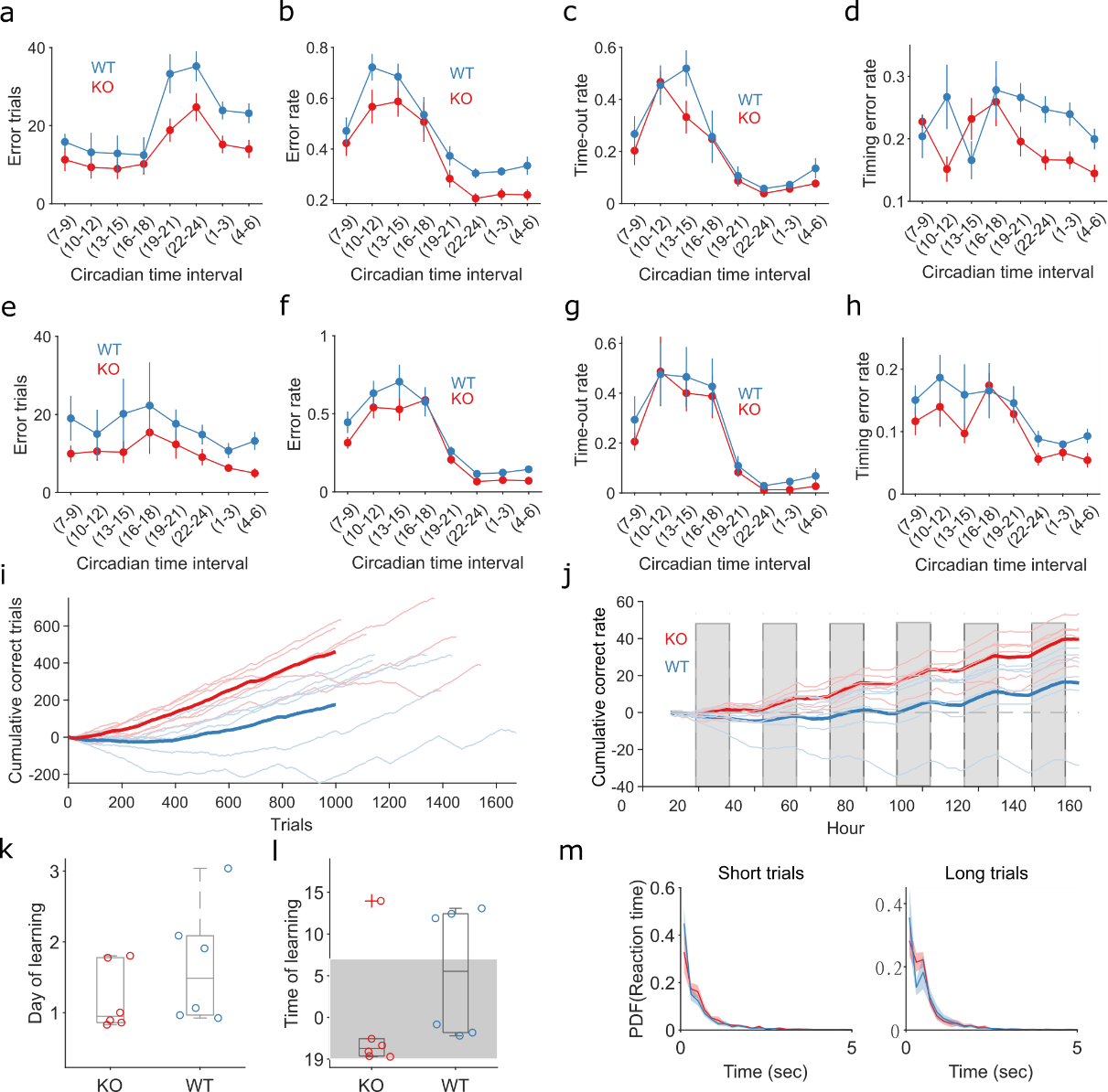
**

**Supplementary Figure 3: TAAR5-KO mice made fewer errors. a.** Absolute number of error trials over the circadian cycle during the first week of training. Values show mean ± SEM as per panels b-h. (2-way ANOVA: significant effect of time p<0.005, F=9.06 and group, p<0.005, F=17.66). **b.** Error rate over the circadian cycle for interval of three hours. 2-way ANOVA significant effect of time p<0.005, F=23.87 and group, p<0.005, F=14.54. **c.** Time-out rate over the circadian cycle for the first week of training. 2-way ANOVA significant effect of time, p<0.005, F=19.66, no effect of group p= 0.08, F=3.03. **d.** Timing error rate during the first week of training along the circadian cycle. 2-way ANOVA no effect of time p=0.07, F=1.93, significant effect of group, p=0.005, F= 8.11. **e.** Similar to panel a for the second week of training. 2-way ANOVA no effect of time p=0.43, F=1, significant effect of group, p=0.006, F=7.82. **f.** Similar to panel b for the second week of training. 2-way ANOVA significant effect of time, p<0.005, F=34.42 and group, p=0.008, F=7.32. **g.** Similar to panel c for the second week of training. 2-way ANOVA significant effect of time, p<0.005, F= 14, no effect of group p=0.32, F=1. **h.** Similar to panel d. 2-way ANOVA significant effect of time, p<0.005, F=5.23 and group, p=0.02, F=5.57. **i.** Cumulative correct trials over the first week of training. Solid lines are group average. Each light color curve is a subject. **j.** Cumulative correct rate for each hour of the first week of training. Solid lines are group average. Each light color curve is a subject. Vertical grey boxes identify the dark phases. **k**. Boxplot distribution of day of learning for WT and KO mice. Each dot is a subject. The central mark in the boxplot indicates the median, the box indicate the interquartile interval and the whiskers extend to the extreme data points not considered outliers (red cross). No difference between groups (Kolmogorov_Smirnov test p=0.31). **l.** Boxplot distribution of the time of learning. Each circle is a subject. Kolmogorov_Smirnov test showed significant difference between group, p = 0.01. **m.** Mean ± SEM probability distribution function of reaction times for each group.
